# Language prediction in monolingual and bilingual speakers: an EEG study

**DOI:** 10.1038/s41598-024-57426-y

**Published:** 2024-03-21

**Authors:** Mohammad Momenian, Mahsa Vaghefi, Hamidreza Sadeghi, Saeedeh Momtazi, Lars Meyer

**Affiliations:** 1 Department of Chinese and Bilingual Studies, The Hong Kong Polytechnic University, CF705, Hung Hom, Kowloon, Hong Kong; 2 Department of Electrical Engineering, Shiraz Branch, Islamic Azad University, Shiraz, Iran; 3 Department of Computer Engineering, Amirkabir University of Technology, Tehran, Iran; 4 Max Planck Institute for Human Cognitive and Brain Sciences, Leipzig, DE Germany; 5 Research Institute for Smart Ageing, The Hong Kong Polytechnic University, Hong Kong, Hong Kong

**Keywords:** Language, Perception

## Abstract

Prediction of upcoming words is thought to be crucial for language comprehension. Here, we are asking whether bilingualism entails changes to the electrophysiological substrates of prediction. Prior findings leave it open whether monolingual and bilingual speakers predict upcoming words to the same extent and in the same manner. We address this issue with a naturalistic approach, employing an information-theoretic metric, surprisal, to predict and contrast the N400 brain potential in monolingual and bilingual speakers. We recruited 18 Iranian Azeri-Persian bilingual speakers and 22 Persian monolingual speakers. Subjects listened to a story in Persian while their electroencephalogram (EEG) was recorded. Bayesian item-level analysis was used. While in monolingual speakers N400 was sensitive to information-theoretic properties of both the current and previous words, in bilingual speakers N400 reflected the properties of the previous word only. Our findings show evidence for a processing delay in bilingual speakers which is consistent with prior research.

## Introduction

Language comprehension is a challenging task because the listener needs to combine representations at different levels (sound, word, sentence, and discourse) under time pressure; they also need to continuously check their current internal model against the physical–acoustic context. Semantic prediction could be beneficial for this process by accelerating decision-making, helping with disambiguation of competing words and reducing memory demands^1^. Semantic prediction is the process of constantly trying to predict the next words as the sentence unfolds.

One of the first behavioural studies on prediction was designed by Altmann and Kamide^2^ using a visual world paradigm, although studies on the effect of semantic context on language processing date back to 1960s (see^3^). Participants were presented with sentences such as ‘The boy will eat the cake’ and ‘The boy will move the cake’. They looked more often at the image of ‘cake’ in the former sentence than ‘cake’ in the latter sentence. This effect was replicated in several studies showing that participants pre-activate the words based on their prior world and semantic knowledge.

In the psycholinguistic literature on linguistic prediction, the N400 is a well-documented event-related potential (ERP; see^4^ for a review). The amplitude of the N400 is reduced for expected words when compared to less-expected words. In an EEG study, Delong, et al.^5^ designed sentences where a target word was either highly plausible or not plausible based on offline cloze probability norming studies. One example is the sentence ‘The day was breezy so the boy went outside to fly’ where ‘a kite’ was judged to have higher plausibility than ‘an airplane’. Since ‘a’ and ‘an’ are not semantically different and are both equally easy to integrate, the difference in N400 amplitude between the two conditions was hypothesized to be an index of prediction. The N400 amplitude had a negative correlation with cloze plausibility ratings at both the article and noun levels. Although this study is about top-down effects across domains (i.e., a semantic prediction facilitating phonetic–phonological processing), it still shows how N400 could be interpreted in the context of predictive coding.

Prior literature is unclear about how speaking multiple languages can affect semantic prediction. Most studies have reported that L2 speakers can predict the upcoming words (see^6,7^ with the differences between L1 and L2 speakers being quantitative, not qualitative^8^. L1 refers to participants’ first language or mother tongue, and L2 refers to the second language. For instance, in Chun and Kan’s study^9^, L2 speakers engaged into predictive processing like L1 listeners, however, L2 speakers’ predictions started later than L1 speakers. In another study Dijkgraaf, et al.^10^ found similar effects in L1 and L2 of bilingual speakers, but semantic representations were weaker and slower in L2 during predictions. The quantitative differences arise due to several factors such as linguistic properties of L1^11^, task-specific effects^8^, lack of cognitive resources in L2 speakers^7,9^, individual differences^8,12^, difficulty level of the context^13,14^, competitive nature of bilingual lexical access^15,16^ and lower L2 proficiency and exposure^11,16,17^. The question is no longer whether L2 speakers predict upcoming words, but what factors and circumstances influence L2 predictive processing^18^.

The majority of previous studies have used a very impoverished context to study how predictions occur (see^19^). Studies with a naturalistic task are not many. One challenge is that traditional ERP analysis cannot be applied to naturalistic settings. In a naturalistic task, the length of the words is different from each other which makes the averaging technique usually used somehow impractical^20^. Novel approaches to analyzing ERP data such as deconvolution-based techniques are now available (see^21^). Statistical power and effect size are usually altered in traditional ERP studies due to limited number of stimuli. Using naturalistic tasks, the problem with number of stimuli is less severe and the findings are more generalizable^22^. Traditionally, human ratings such as cloze probabilities have been used to operationalize prediction. However, recently information metrics such as surprisal which are created by language models have been shown to predict N400 more robustly than human ratings^23^. Unlike cloze probabilities which are sensitive to both speakers’ linguistic and non-linguistic (world) knowledge, information metrics only reflect linguistic patterns^24^, therefore minimizing the effects of world knowledge.

In this study, we recruited 40 monolingual and bilingual participants to listen to 30 min of a natural story in Persian (Alice in Wonderland). We extracted a complexity metric such as surprisal using a Persian gpt2 language model. Surprisal measures the degree of unexpectedness upon reading or hearing a word; high surprisal means the word was unexpected^25–27^. Our purpose is to leverage this metric for assessing whether the N400 differs between monolingual and bilingual speakers, thus shedding light on the research question whether lexical-semantic prediction is similar across these groups. We expect a delay in the effect of surprisal on the N400 in bilingual speakers because they are usually slower than monolingual speakers in lexical retrieval. The slower retrieval in bilingual speakers is explained in terms of weaker connections between semantic and phonological representations of the words^28,29^ or competition among the words in the mental lexicon^30,31^.

## Results

**Figure 1:**
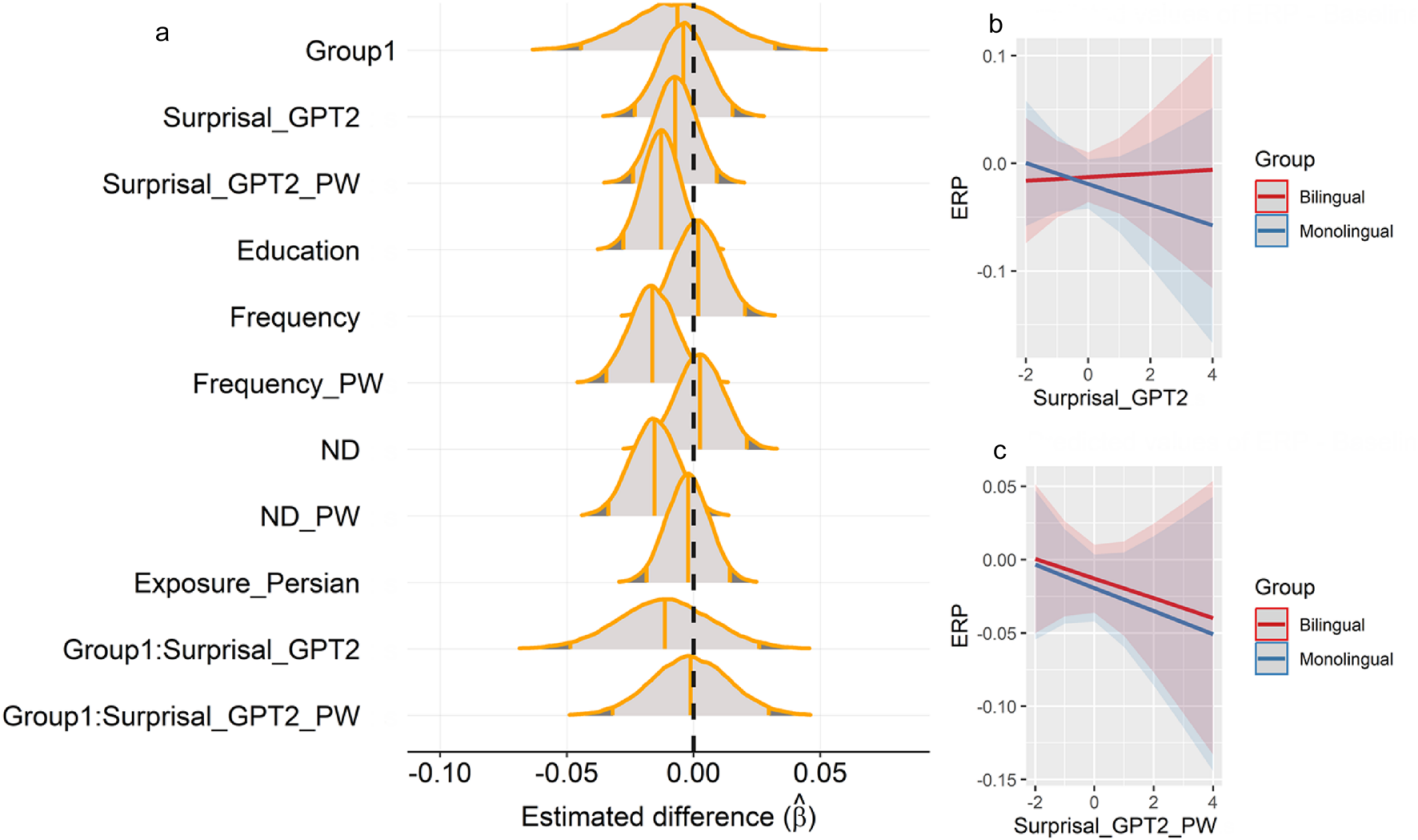
Posterior predictions and effects of Surprisal and Surprisal_PW on average ERP across the two groups. Variables are standardized. (**a**) The area in the middle of each distribution shows the 95% credible interval. The vertical line in the middle of shaded area is posterior mean. Group is deviation coded (− 0.5 and 0.5). (**b**) Surprisal of the current word has different effects on ERP in both groups. (**c**) Spill-over effect is shown here where surprisal of the previous word predicted the ERP of the current word. Higher surprisal of the previous word results in more negative ERP in both groups. The shades are confidence intervals with outer probability = 0.89 and inner probability = 0.50. Negative is plotted down.

ERP amplitude is slightly more negative in monolingual speakers than bilingual speakers in the selected channels. In the monolingual group, posterior predictions suggest a moderate effect for surprisal of the current word; higher surprisal equals more negative amplitude (Credible Interval (CI) = − 0.04, 0.02; see Fig. 1b). On the other hand, the ERP of the current word observed in bilingual speakers does not reflect properties such as surprisal (CI = − 0.03, 0.03; See Fig. 1b). The spill-over effects of surprisal are similar for both monolingual (CI = -0.03, 0.01) and bilingual speakers (CI = − 0.03,0.02) meaning that processing of the current word is influenced by the surprisal of the previous word (See Fig. 1c), although CIs suggest an stronger effect in the monolingual group. When it comes to other variables in the model, there is strong evidence for effects of education, frequency of the previous word, and neighborhood density (ND) of the previous word (see Fig. 1a). The full results of the model are presented in Table 1 (see Fig. 1a for the posterior distributions of the same model).

**Table 1:** Results of the Bayesian analysis.

Predictors	Estimate	95% CI	R hat	Bulk ESS	Tail ESS
Intercept	− 0.02	− 0.03, − 0.00	1.00	67,427	27,829
Group	− 0.01	− 0.04, 0.03	1.00	57,334	28,612
Surprisal_GPT2	− 0.00	− 0.02, 0.02	1.00	45,364	31,349
Surprisal_GPT2_PW	− 0.01	− 0.02, 0.01	1.00	59,320	29,574
Education	− 0.01	− 0.03, 0.00	1.00	58,574	31,344
Frequency	0.00	− 0.02, 0.02	1.00	64,687	28,427
Frequency_PW	− 0.02	− 0.03, 0.00	1.00	67,064	28,250
ND	0.00	− 0.02, 0.02	1.00	70,128	30,027
ND_PW	− 0.02	− 0.03, 0.00	1.00	65,601	27,850
Exposure to Persian	− 0.00	− 0.02, 0.01	1.00	59,732	31,370
Group: Surprisal_GPT2	− 0.01	− 0.05, 0.03	1.00	42,495	29,089
Group:Surprisal_GPT2_PW	− 0.00	− 0.03, 0.03	1.00	54,271	30,130
Random effects					
Item (Intercept)	0.13	0.011, 0.15	1.00	17,138	25,077
Group	0.25	0.21, 0.30	1.00	16,694	22,955
Cor (Group, Intercept)	0.92	0.79, 0.99	1.00	7756	14,341
Subject (Intercept)	0.01	0.00, 0.02	1.00	22,721	20,810
Surprisal_GPT2	0.04	0.02, 0.06	1.00	14,202	18,901
Surprisal_GPT2_PW	0.03	0.00, 0.05	1.00	8589	9612

Subjects: 38, Items: 4150, number of observations: 157,455. Group is deviation coded. ESS (Effective Sample Size).

To do model diagnostics, we first looked at trace plots. Visual inspection of the trace plots revealed that the chains converged. This was further confirmed by looking at between-to within-chain variances (R hat). R hat values were all 1 showing convergence with no issues (See Table 1). The posterior predictive checking showed that the simulated distribution was similar to the observed data.

## Discussion and conclusion

In this study, we used an information-theoretic approach to assess whether monolingual and bilingual speakers differ in the electrophysiological substrate of semantic prediction. A deep neural network was used to estimate word-by-word surprisal from Persian texts. We extracted neural responses for each word which was later used in Bayesian mixed effects analysis. While our analysis revealed a difference between monolingual and bilingual speakers in the effect of surprisal on the N400, a similar spill-over effect in both groups was observed suggesting that the difference between the two groups could be a matter of processing time only.

Our findings are consistent with those studies which have shown the difference between monolingual and bilingual speakers is only ‘quantitative’. These studies show that bilingual speakers engage in predictive processing, but there is usually a delay observed in the effect^9,32,33^. This delay in bilingual speakers could be explained in terms of a retrieval or ‘processing deficit’^34^. The source of this deficit could be either at the correspondence between semantic and phonological representations of words^35^ or interference caused by competition among the words in the lexicon^30,31^. The mechanism underlying this deficit could be different depending on each explanation, but the outcome which is a delay in lexical retrieval and thus semantic prediction will be the same no matter which mechanism is in charge.

In addition to the retrieval deficit account, we think our findings could be interpreted based on a prediction error account as well. Based on this account, bilingual speakers should demonstrate stronger effects for surprisal (as an index of prediction error) in comparison with monolingual speakers. In several studies, bilingual speakers show improved performance in domain-general conflict resolution and attentional control (see^36,37^). However, in our study this reported domain-general advantage in bilingual speakers did not modify our findings. Future research is needed to study how attentional control could affect resolving prediction errors in bilingual speakers.

The spill-over effect is consistent with some previous studies. Smith and Levy^38^ showed that the surprisal of a word affected both that word and the words immediately following it (see^39^). This effect interacted with the type of the task. When it came to eye-tracking, the surprisal effect was immediately shown on the same word and the next word. However, when it came to self-paced reading, the effect was not so immediate. It started on the next word and was there through the third word. In the bilingual speakers of our study, it was the surprisal of the previous word which affected N400 of the current word.

Some studies show that for prediction effects to be evident in L2 processing, speakers should be given more time (See^40^). Predictive effects were absent particularly when the prediction context was challenging such as when the speed of delivery was high or the gap between the words was too short^13,14^. We used a naturalistic task in our study which used a normal pace of delivery. The gap between the words was not manipulated. The naturalistic property of our task could explain the spill-over effect in the bilingual group. It seems that bilingual speakers show a delay to the properties of words such as surprisal. Future studies could be done on naturalistic tasks with a lower rate of delivery.

We know that bilingual experience has been shown to affect predictive processing (see^11,16,17^). In our study, the bilingual speakers were balanced speakers with a high degree of exposure to their L2 comparable to L1 of monolingual speakers. Indeed, the bilingual participants reported more exposure to their L2 than L1. This was because Azerbaijani, their L1, is mainly spoken in north-western regions of Iran. The participants of this study were recruited from Tehran where the daily language is Persian. We, therefore, don’t think the bilingual experience could explain our findings. Moreover, since we did not control the language-specific properties for Azeri and Persian in this study, we think our results generalize to this pair only or a typologically similar pair.

The finding that frequency and orthographic neighbourhood density of the current word did not have any effects on N400 amplitude is not consistent with previous studies. Several studies have shown that both frequency and neighbourhood density could modulate the N400 amplitude: the lower the frequency and the higher the neighbourhood density of the word is, the larger the N400 amplitude will be^41,42^. One explanation for this finding could be that the effects of frequency and neighbourhood density are diminished when surprisal is in the model (see^43,44^). Shain^45^ showed that frequency and predictability had effects on reading times in isolation, but the effect of frequency disappeared once predictability and frequency were both in the same model. However, if this explanation was true, we should have observed the same effect in the frequency and neighbourhood density of the previous word. Future studies could create low frequency and high frequency lists of stimuli (same with neighbourhood density) and look at the interaction between frequency and surprisal. Our design did not allow us to do so because we used a naturalistic task and we did not want to dichotomize a continuous variable.

Most previous studies used information-theoretic metrics with monolingual speakers (See^46^). Our study showed that one such metric such as surprisal could predict N400 in bilingual speakers as well. Although information-theoretic metrics have been shown to link cognitive theories with neural signals^47^, our study only showed a statistical relationship. Further studies need to be conducted explaining why this relationship exists^25^. The metrics used in this study were only extracted from GPT2 language model since other big language models such as LlaMA, Falcon, BARD, GPT 3.5, BLOOM, and BERT were not available in Persian. This is another limitation. At the moment, our findings need to be interpreted with caution.

In this study, we did not control cognitive functions such as working memory, processing speed, and inhibition. Huettig and Janse^12^ showed that individual differences in working memory and processing speed could predict anticipatory processing. This is more important in bilingual speakers because several studies reveal that these speakers have trouble allocating enough resources for the prediction of words^7,8,48^. Future studies could investigate whether bilingual speakers with high working memory capacity and processing speed predict words similarly to those who have lower levels of working memory capacity and processing speed.

## Methods

### Participants

We recruited 18 Iranian Azeri-Persian bilingual speakers (Female = 9, age mean = 27.22, L1 exposure = 35.27%, L2 exposure = 58.88%, L2 AOA = 5.83, education mean = 18.55 years) and 22 Persian monolingual speakers (Female = 11, age mean = 26.09, L1 exposure = 87.72%, L2 exposure = 16.68%, education mean = 16.27 years). Data from two bilingual participants was discarded due to high levels of noise. Participants’ language history was documented using the Language Experience and Proficiency Questionnaire (LEAP-Q)^49^. Most of our participants were university students. Bilingual speakers were recruited from the city of Tehran where Persian is spoken as the medium of communication among people which is why bilingual participants reported more exposure to their L2 than L1. All speakers in this study rated their proficiency in Persian or/and Turkish to be at least 7 out of 10. The L2 exposure in monolingual speakers refers to the exposure to English. In Iran, students learn English from secondary school which is mainly limited to vocabulary and grammar. The monolingual speakers reported their proficiency in English less than 4 out of 10. All participants reported they were right-handed^50^. They had no history of neurological diseases. Informed consent was obtained from all participants before the experiment started. Ethical approval was obtained from the Ethics Committee at Iran University of Medical Sciences (IR.IUMS.REC.1397.414). This study was performed in accordance with the Declaration of Helsinki and all other regulations set by the Ethics Committee. This study was conducted at the National Brain Mapping Lab, Tehran, Iran.

### Procedure

Participants listened to 6 chapters (about 30 min) of Alice in Wonderland in Persian while they were looking at a fixation cross. Before they started listening to the story, we checked the audio level for each participant and made sure they could hear clearly. Before the main experiment, we played the 6 chapters to a different group of people to check the speed with which the story was played. The group reported that the speed was at the normal level and easily comprehensible. To make sure the participants were listening to the story, we presented 5 comprehension check questions (yes/no) at the end of each chapter of the story. Answering the questions was not timed. The average response accuracy was 74%.

The EEG equipment used in this study was a 64 channel g.tec machine. The sampling rate was 512 Hz. The software used to present the stimuli was Psychtoolbox.

### EEG data analysis

EEG pre-processing was carried out using EEGLAB^51^, Fieldtrip^52^, as well as customized MATLAB codes (The MathWorks, Inc., Natick, US). We modified the Harvard Automated Preprocessing Pipeline^53^ (HAPPE) to perform EEG preprocessing automatically.

**Figure 2:**
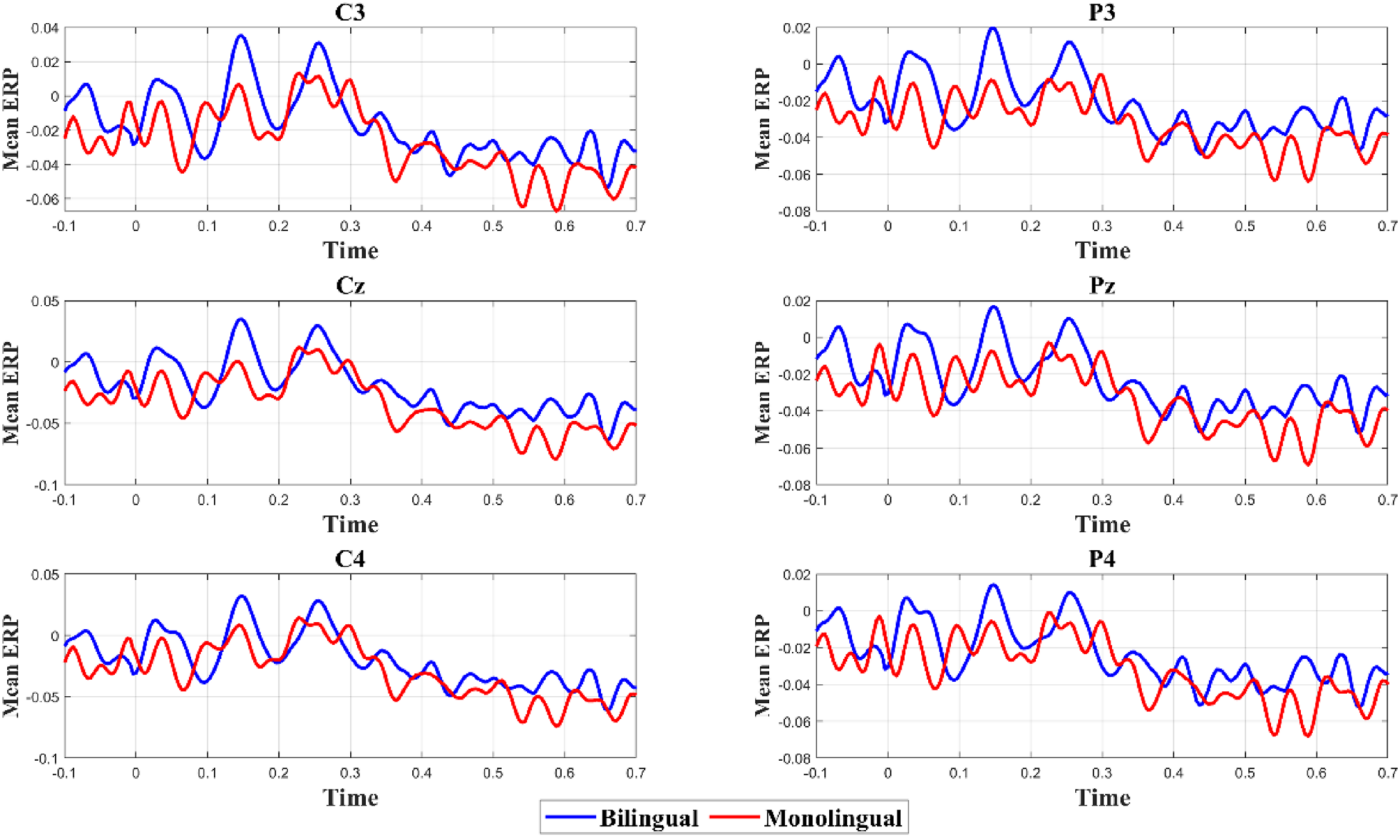
Mean ERP for 6 channels of interest for both groups after low pass filtering and demeaning. Zero on the x axis is aligned to the onset of the target word. No baseline correction was applied here.

Data from all channels was re-referenced to an average (i.e., A2 and A1 re-reference). By evaluating the normed joint probability of the average log power from 1 to 500 Hz, we discovered the noisy channels and removed those that exceeded a 3 SD threshold from the mean. Electrical line noise at 50 Hz was removed using ZapLine of the NoiseTools toolbox^54^. The signal was also passed through a high-pass finite impulse response filter with a cutoff frequency of 0.1 Hz before we conducted ICA. To remove artifacts such as eye and muscle artifacts, high-amplitude artifacts (e.g., blinks), and signal discontinuities (e.g., electrodes losing contact with the scalp), a wavelet-enhanced ICA^55^ (W_ICA) was used. Following W_ICA, the Multiple Artifact Rejection Algorithm (MARA), a machine learning algorithm that evaluates the ICA-derived components, was used to perform automated component rejection^56^. The data for bad channels was interpolated with spherical splines for each segment. At the end of the pre-processing stage, data was down-sampled to 250 Hz, filtered with a 30 Hz Butterworth low-pass filter, and demeaned (See Fig. 2 for mean ERP).

After the preprocessing step, the pipeline suggested in the Unfold toolbox^21^ was used. First, the model of interest was specified, and the design matrix was generated. All onsets of the target words from the story were defined as events in the continuous EEG data. We defined an interval (− 250 ms to 800 ms) around each target word using an only-intercept model (ERP ~ 1) in line with regression-based ERP (rERP) approaches^57^. The time basis function used was the stick-function approach which is the default option in Unfold. We replaced the intervals containing EEG artifacts with zero because removing these intervals could influence model estimation in continuous data^57^. In the next stage, the regression model was solved using LSMR^58^ which is an iterative algorithm for sparse least-squares problems. At the final stage, we extracted betas for all words for the interval of interest (300 to 500) which are used later for item level analysis.

For baseline correction, we used the approach recommended by Alday^59^. Unlike the traditional approach of baseline correction, in this approach baseline is not treated the same for each participant and item meaning each participant and item can have a unique baseline. This is important in naturalistic data because there is no baseline in the traditional sense before each word; words come after one another without any interval. In our study, we extracted the baseline for each item (− 100–0 ms) and then subtracted from our time window of analysis (300–500 ms) (see^60^). Applying baseline correction after the deconvolution is till acceptable^21,60,61^.

### Language models

Surprisal, known as a complexity metric, and human based plausibility ratings have been used frequently to study predictive processing (see^62^). One concern with cloze probabilities is, however, that they are usually averaged across items, therefore cannot capture the trial-specific variance^26^. Surprisal measures the degree of unexpectedness upon reading or hearing a word; high surprisal means the word was clearly unexpected. Surprisal is a backward-looking measure^25^. Surprisal could, therefore, be used to study how prediction errors could be resolved^26,27^. Metrics estimated by language models such as GPT-3 have been argued to predict N400 much better then human judgements^63^.

A GPT model which was pre-trained on Persian language, called bolbolzaban/gpt2-persian (https://www.bolbolzaban.com), is used for our goal. The context size of this language model was reduced from 1024 (in the original model in English) to 256 to make the training of the model affordable. In this model, all English words and numbers were replaced with special tokens and only standard Persian alphabet was used as part of input text. This model included about 328 million parameters. In this study, we focused only on surprisal. Since Persian GPT-3 has not been released as open source, we use GPT-2 language model in this research study.

Hale^64^ first introduced the surprisal theory. The surprisal theory of incremental language processing characterizes the lexical predictability of a token w_t_ in terms of a surprisal value which is the negative logarithm of the conditional probability of a token given its preceding context, -LogP (w_t_|w_t-1_, …, w_0_). The higher the surprisal values the smaller conditional probabilities; i.e., tokens that are less predictable are more surprising to the language user and are harder to process as a result^65^. In this paper, the conditional probability of a given token is obtained from the GPT-2 model, and then the negative value of its logarithm is considered as surprisal.

In addition to surprisal, we included the frequency of each word and neighborhood density (ND) in the analysis following the recommendation by Sassenhagen^66^. Word frequency and ND have been shown to be correlated with neural activity (see^4^). Neighborhood density refers to the number of phonologically similar words in the lexicon and is often calculated by determining the number of words that are created by adding, deleting, or substituting a single character in a given word^67^. For example, the word “sit” has 36 neighbors including “spit”, “it”, and “hit”. Words with a high number of neighbors are said to reside in dense neighborhoods, whereas those with few neighbors reside in sparse neighborhoods. In this study, a dataset extracted from a Persian news website called Bartarinha was used to find the ND of each word. To calculate the frequency of each word, we used Hamshahri2 corpus^68^. This is one of the biggest corpora available in Persian consisting of several types of genres and documents.

### Bayesian analysis

We used Bayesian linear mixed effects modelling to analyze the data. The package brms was used^69^. We used four chains, four cores, 10,000 iterations, 1000 of which were warm-ups. Since N400 has a centroparietal distribution, the dependent variable was the average scalp potential over six channels Cz, C3, C4, Pz, P3, and P4^70^ within a time window of 300–500 ms. All continuous predictor variables were centered (mean = 0, SD = 1). Group (monolingual and bilingual) was deviation coded (− 0.5 and 0.5) at first.

Fixed effects included group (bilingual vs. monolingual), exposure to Persian language, and the following properties of the current word (CW) and the immediate previous word (PW) such as surprisal, frequency, and neighborhood density (ND). The interaction between group and surprisal of the CW and PW was included in the model. For the random effects structure, we included words and participants as random intercepts. Group was used as a by-word random slope and surprisal of the CW and PW was used as by-participant random slopes. There were no correlation parameters for the by-participant random slopes to avoid any convergence problems. The following is the structure of the full model:

$$\begin{gathered} {\text{Amplitude }}\sim {\text{ Group }}\left( {{\text{Surprisal}}\_{\text{GPT2 }}\, + {\text{ Surprisal}}\_{\text{GPT2}}\_{\text{PW}}} \right) \, + {\text{ Frequency }} \hfill \\ + {\text{ ND }} + {\text{ Exposure to Persian }} + {\text{ Frequency}}\_{\text{PW }} + {\text{ ND}}\_{\text{PW }} + \, \left( {{1} + {\text{ Group}}|{\text{Word}}} \right) \, \hfill \\ + \left( {{1} + {\text{ Surprisal}}\_{\text{GPT2 }}+ {\text{ Surprisal }}\_{\text{GPT2}}\_{\text{PW}}||{\text{Participant}}} \right) \hfill \\ \end{gathered}$$

We then fitted two separate models with each level of group (monolingual or bilingual) as the reference level in the model. We dummy coded the group variable to see what the effects of the other predictor variables are within each group of participants in this study. To do model diagnostics, we used the between-to within-chain variances (R hat) and did a visual inspection of the chains. R hat values should be 1 or close to show that chains converged. We also used posterior predictive (PP) checks to see if the data fitted the model properly or not^71,72^.

We used regularizing or weakly informative priors. The advantage of using weakly informative priors is that they produce stable inferences^73^. We use posterior probability, mean estimates and Bayesian credible intervals (CI) to report the results. We used the following priors in brms based on Nicenboim, et al.^73^.

$$\begin{gathered} {\text{reg}}\_{\text{priors}} < {-}{\text{ c }}\left( {{\text{prior }}\left( {{\text{normal }}\left( {0,{ 1}0} \right),{\text{ class}} = {\text{Intercept}}} \right),} \right. \hfill \\ \quad \quad \quad \quad \quad {\text{prior }}\left( {{\text{normal }}\left( {0,{ 1}} \right),{\text{ class}} = {\text{b}}} \right), \hfill \\ \quad \quad \quad \quad \quad {\text{prior }}\left( {{\text{normal }}\left( {0,{ 1}0} \right),{\text{ class}} = {\text{sd}}} \right), \hfill \\ \quad \quad \quad \quad \quad {\text{prior }}\left( {{\text{normal }}\left( {0,{1}0} \right),{\text{ class}} = {\text{sigma}}} \right), \hfill \\ \left. {\quad \quad \quad \quad \quad {\text{prior }}\left( {{\text{lkj }}\left( {2} \right),{\text{ class}} = {\text{cor}}} \right)} \right). \hfill \\ \end{gathered}$$

## Data Availability

The datasets generated during and analysed during the current study are available in the OSF repository with 10.17605/OSF.IO/JHPX8.

## References

[1] Kutas M, DeLong KA, Smith NJ (2011). Predictions in the Brain Using Our Past to Generate a Future.

[2] Altmann GTM, Kamide Y (1999). Incremental interpretation at verbs: Restricting the domain of subsequent reference. Cognition.

[3] Miller GA, Heise GA, Lichten W (1951). The intelligibility of speech as a function of the context of the test materials. J. Exp. Psychol..

[4] Kutas M, Federmeier KD (2011). Thirty years and counting: finding meaning in the N400 component of the event-related brain potential (ERP). Annu. Rev. Psychol..

[5] Delong KA, Urbach TP, Kutas M (2005). Probabilistic word pre-activation during language comprehension inferred from electrical brain activity. Nat. Neurosci..

[6] Dijkgraaf A, Hartsuiker RJ, Duyck W (2017). Predicting upcoming information in native-language and non-native-language auditory word recognition. Biling. Lang. Cognit..

[7] Foucart A, Martin CD, Moreno EM, Costa A (2014). Can bilinguals see it coming? Word anticipation in L2 sentence reading. J. Exp. Psychol.: Learn. Memory Cognit..

[8] Kaan E (2014). Predictive sentence processing in L2 and L1. Linguist. Approaches Biling..

[9] Chun E, Kaan E (2019). L2 Prediction during complex sentence processing. J. Cult. Cognit. Sci..

[10] Dijkgraaf A, Hartsuiker RJ, Duyck W (2019). Prediction and integration of semantics during L2 and L1 listening. Lang. Cognit. Neurosci..

[11] Dussias PE, Valdés Kroff JR, Guzzardo Tamargo RE, Gerfen C (2013). When gender and looking go hand in hand grammatical gender processing In L2 Spanish. Stud. Second Lang. Acquis..

[12] Huettig F, Janse E (2016). Individual differences in working memory and processing speed predict anticipatory spoken language processing in the visual world. Lang. Cognit. Neurosci..

[13] Grüter T, Lew-Williams C, Fernald A (2012). Grammatical gender in L2: A production or a real-time processing problem?. Second Lang. Res..

[14] Lew-Williams C, Fernald A (2010). Real-time processing of gender-marked articles by native and non-native Spanish speakers. J. Mem. Lang..

[15] Peters R, Grüter T, Borovsky A (2018). Vocabulary size and Native Speaker self-identification influence flexibility in linguistic prediction among adult bilinguals. Appl. Psycholinguist.

[16] van Bergen G, Flecken M (2017). Putting things in new places: Linguistic experience modulates the predictive power of placement verb semantics. J. Mem. Lang..

[17] Hopp H (2013). Grammatical gender in adult L2 acquisition: Relations between lexical and syntactic variability. Second Lang. Res..

[18] Kaan, E. & Grüter, T. in *Prediction in second language processing and learning* (eds E. Kaan & T Grüter) 2–24 (John Benjamins Publishing Company, 2021).

[19] Huettig F (2015). Four central questions about prediction in language processing. Brain Res..

[20] Alday PM, Schlesewsky M, Bornkessel-Schlesewsky I (2017). Electrophysiology reveals the neural dynamics of naturalistic auditory language processing: Event-related potentials reflect continuous model updates. eneuro.

[21] Ehinger BV, Dimigen O (2019). Unfold: An integrated toolbox for overlap correction, non-linear modeling, and regression-based EEG analysis. PeerJ.

[22] Hamilton LS, Huth AG (2020). The revolution will not be controlled: Natural stimuli in speech neuroscience. Lang. Cogn. Neurosci..

[23] Michaelov, J. A., Coulson, S. & Bergen, B. K. So cloze yet so far: N400 amplitude is better predicted by distributional information than human predictability judgements. *arXiv* (2021).

[24] Frank SL, Otten LJ, Galli G, Vigliocco G (2015). The ERP response to the amount of information conveyed by words in sentences. Brain Lang..

[25] Armeni K, Willems RM, Van Den Bosch A, Schoffelen J-M (2019). Frequency-specific brain dynamics related to prediction during language comprehension. NeuroImage.

[26] Kuperberg GR, Jaeger TF (2016). What do we mean by prediction in language comprehension?. Lang. Cognit. Neurosci..

[27] Willems RM, Frank SL, Nijhof AD, Hagoort P, Van Den Bosch A (2016). Prediction during natural language comprehension. Cerebral Cortex.

[28] Gollan TH, Montoya RI, Fennema-Notestine C, Morris SK (2005). Bilingualism affects picture naming but not picture classification. Mem. Cognit..

[29] Gollan TH, Montoya RI, Cera C, Sandoval TC (2008). More use almost always a means a smaller frequency effect: Aging, bilingualism, and the weaker links hypothesis. J. Mem. Lang..

[30] Abutalebi J, Green DW (2008). Control mechanisms in bilingual language production: Neural evidence from language switching studies. Lang. Cognit. Process..

[31] Green DW (1998). Mental control of the bilingual lexico-semantic system. Biling. Lang. Cognit..

[32] Chun, E., Chen, S., Liu, S. & Chan, A. in *Prediction in Second Language Processing and Learning* (eds E. Kaan & T. Gruter) 69–90 (John Benjamins Publishing Company, 2021).

[33] Schlenter J (2023). Prediction in bilingual sentence processing: How prediction differs in a later learned language from a first language. Biling. Lang. Cognit..

[34] Kaan E, Kirkham J, Wijnen F (2016). Prediction and integration in native and second-language processing of elliptical structures. Biling. Lang. Cogni..

[35] Kroll JF, Gollan TH (2014). The Oxford Handbook of Language. Production OXFORD Library of Psychology.

[36] Bialystok E, Craik FIM (2022). How does bilingualism modify cognitive function? Attention to the mechanism. Psychon. Bull. Rev..

[37] Zirnstein M, van Hell JG, Kroll JF (2018). Cognitive control ability mediates prediction costs in monolinguals and bilinguals. Cognition.

[38] Smith NJ, Levy R (2013). The effect of word predictability on reading time is logarithmic. Cognition.

[39] van Schijndel M, Linzen T (2021). Single-stage prediction models do not explain the magnitude of syntactic disambiguation difficulty. Cognit. Sci..

[40] Fernandez LB, Engelhardt PE, Patarroyo AG, Allen SE (2020). Effects of speech rate on anticipatory eye movements in the visual world paradigm: Evidence from aging, native, and non-native language processing. Q. J. Exp. Psychol. (Hove).

[41] Meade G, Grainger J, Holcomb PJ (2019). Task modulates ERP effects of orthographic neighborhood for pseudowords but not words. Neuropsychologia.

[42] Carrasco-Ortiz H, Midgley KJ, Grainger J, Holcomb PJ (2017). Interactions in the neighborhood: Effects of orthographic and phonological neighbors on N400 amplitude. J. Neurolinguistics.

[43] Fruchter J, Linzen T, Westerlund M, Marantz A (2015). Lexical preactivation in basic linguistic phrases. J. Cognit. Neurosci..

[44] Huizeling E, Arana S, Hagoort P, Schoffelen J-M (2022). Lexical frequency and sentence context influence the brain’s response to single words. Neurobiol. Lang..

[45] Shain, C. (Association for Computational Linguistics).

[46] Brennan JR, Hale JT (2019). Hierarchical structure guides rapid linguistic predictions during naturalistic listening. PLOS One.

[47] Brennan J (2016). Naturalistic sentence comprehension in the brain. Lang. Linguist. Compass.

[48] Grüter T, Rohde H (2021). Limits on expectation-based processing: Use of grammatical aspect for co-reference in L2. Appl. Psycholinguist..

[49] Marian V, Blumenfeld HK, Kaushanskaya M (2007). The language experience and proficiency questionnaire (LEAP-Q): Assessing language profiles in bilinguals and multilinguals. J. Speech, Lang. Hear. Res..

[50] Oldfield RC (1971). The assessment and analysis of handedness: The Edinburgh inventory. Neuropsychologia.

[51] Delorme A, Makeig S (2004). EEGLAB: An open source toolbox for analysis of single-trial EEG dynamics including independent component analysis. J. Neurosci. Methods.

[52] Oostenveld R, Fries P, Maris E, Schoffelen JM (2011). FieldTrip: Open source software for advanced analysis of MEG, EEG, and invasive electrophysiological data. Comput. Intell Neurosci..

[53] Gabard-Durnam LJ, Mendez Leal AS, Wilkinson CL, Levin AR (2018). The harvard automated processing pipeline for electroencephalography (HAPPE): Standardized processing software for developmental and high-artifact data. Front. Neurosci..

[54] de Cheveigné A (2020). ZapLine: A simple and effective method to remove power line artifacts. Neuroimage.

[55] Castellanos NP, Makarov VA (2006). Recovering EEG brain signals: artifact suppression with wavelet enhanced independent component analysis. J. Neurosci. Methods.

[56] Winkler I, Haufe S, Tangermann M (2011). Automatic classification of artifactual ICA-components for artifact removal in EEG signals. Behav. Brain Funct..

[57] Smith NJ, Kutas M (2015). Regression-based estimation of ERP waveforms: I. The rERP framework. Psychophysiology.

[58] Fong DC-L, Saunders M (2011). LSMR: An iterative algorithm for sparse least-squares problems. SIAM J. Sci. Comput..

[59] Alday PM (2019). How much baseline correction do we need in ERP research? Extended GLM model can replace baseline correction while lifting its limits. Psychophysiology.

[60] Dimigen O, Ehinger BV (2021). Regression-based analysis of combined EEG and eye-tracking data: Theory and applications. J. Vis..

[61] Smith NJ, Kutas M (2015). Regression-based estimation of ERP waveforms: II. Non-linear effects, overlap correction, and practical considerations. Psychophysiology.

[62] Hale J (2016). Information-theoretical complexity metrics. Lang. Linguist. Compass.

[63] Michaelov JA, Coulson S, Bergen BK (2022). So cloze yet so Far: N400 amplitude is better predicted by distributional information than human predictability judgements. IEEE Trans. Cognit. Dev. Syst..

[64] Hale, J. in *North American Chapter of the Association for Computational Linguistics.* (Association for Computational Linguistics).

[65] Luong, T., Donnell, T. & Goodman, N. in *Sixth Workshop on Cognitive Aspects of Computational Language Learning.*

[66] Sassenhagen J (2019). How to analyse electrophysiological responses to naturalistic language with time-resolved multiple regression. Lang. Cognit. Neurosci..

[67] Luce PA, Pisoni DB (1998). Recognizing spoken words: The neighborhood activation model. Ear Hear.

[68] AleAhmad A, Amiri H, Darrudi E, Rahgozar M, Oroumchian F (2009). Hamshahri: A standard Persian text collection. Knowl.-Based Syst..

[69] Bürkner P-C (2017). brms: An R package for Bayesian multilevel models using stan. J. Stat. Softw..

[70] Nieuwland MS (2018). Large-scale replication study reveals a limit on probabilistic prediction in language comprehension. eLife.

[71] Gabry J, Simpson D, Vehtari A, Betancourt M, Gelman A (2019). Visualization in Bayesian workflow. J. Royal Statist. Soc. Series A: Statist. Soc..

[72] Schad DJ, Betancourt M, Vasishth S (2021). Toward a principled Bayesian workflow in cognitive science. Psychol. Methods.

[73] Nicenboim B, Vasishth S, Rösler F (2020). Are words pre-activated probabilistically during sentence comprehension? Evidence from new data and a Bayesian random-effects meta-analysis using publicly available data. Neuropsychologia.

